# A Safe and Stable Neonatal Vaccine Targeting GAPDH Confers Protection against Group B Streptococcus Infections in Adult Susceptible Mice

**DOI:** 10.1371/journal.pone.0144196

**Published:** 2015-12-16

**Authors:** Joana Alves, Pedro Madureira, Maria Teresa Baltazar, Leandro Barros, Liliana Oliveira, Ricardo Jorge Dinis-Oliveira, Elva Bonifácio Andrade, Adília Ribeiro, Luís Mira Vieira, Patrick Trieu-Cuot, José Alberto Duarte, Félix Carvalho, Paula Ferreira

**Affiliations:** 1 ICBAS, Instituto de Ciências Biomédicas Abel Salazar, Universidade do Porto, Porto, Portugal; 2 Instituto de Investigação e Inovação em Saúde, Universidade do Porto, Porto, Portugal; 3 IBMC, Instituto de Biologia Molecular e Celular, Universidade do Porto, Porto, Portugal; 4 REQUIMTE, Laboratório de Toxicologia, Faculdade de Farmácia, Universidade do Porto, Porto, Portugal; 5 Department of Legal Medicine and Forensic Sciences, Faculty of Medicine, University of Porto, Porto, Portugal; 6 IINFACTS, Institute of Research and Advanced Training in Health Sciences and Technologies, Department of Sciences, Advanced Institute of Health Sciences, North (ISCS-N), CESPU, CRL, Gandra, Portugal; 7 Institut Pasteur, Unité de Biologie des Bactéries Pathogènes à Gram-Positif, Centre National de la Recherche Scientifique (CNRS ERL 3526), Paris, France; 8 CIAFEL, Faculdade de Desporto, Universidade do Porto, Porto, Portugal; Centers for Disease Control & Prevention, UNITED STATES

## Abstract

Group B *Streptococcus* (GBS), a commensal organism, can turn into a life-threatening pathogen in neonates and elderly, or in adults with severe underlying diseases such as diabetes. We developed a vaccine targeting the GBS glyceraldehyde-3-phosphate dehydrogenase (GAPDH), a glycolytic enzyme detected at the bacterial surface, which was proven to be effective in a neonatal mouse model of infection. Since this bacterium has emerged as an important pathogen in non-pregnant adults, here we investigated whether this vaccine also confers protection in an adult susceptible and in a diabetic mouse model of infection. For immunoprotection studies, sham or immunized adult mice were infected with GBS serotype Ia and V strains, the two most prevalent serotypes isolated in adults. Sham and vaccinated mice were also rendered diabetic and infected with a serotype V GBS strain. For toxicological (pre-clinical) studies, adult mice were vaccinated three times, with three concentrations of recombinant GAPDH adjuvanted with Allydrogel, and the toxicity parameters were evaluated twenty-four hours after the last immunization. For the stability tests, the vaccine formulations were maintained at 4°C for 6 and 12 months prior immunization. The results showed that all tested doses of the vaccine, including the stability study formulations, were immunogenic and that the vaccine was innocuous. The organs (brain, blood, heart, and liver) of vaccinated susceptible or diabetic adult mice were significantly less colonized compared to those of control mice. Altogether, these results demonstrate that the GAPDH-based vaccine is safe and stable and protects susceptible and diabetic adult mice against GBS infections. It is therefore a promising candidate as a global vaccine to prevent GBS-induced neonatal and adult diseases.

## Introduction

*Streptococcus agalactiae*, or Group B *Streptococcus* (GBS), is the leading cause of life-threatening bacterial infections in newborns [1]. In the past three decades, this bacterium have emerged as a major cause of invasive infections in non-pregnant adults, mainly in individuals with more than 65 years old or with underlying medical conditions [2–5]. Diabetes mellitus appears as the most common predisposition for GBS bacteremia in this group [2, 5]. The case fatality rates are higher in adults than in neonates [2, 6]. Eighty percent of human GBS isolates are resistant to tetracycline and it has been recently proposed that the widespread use of this antibiotic from 1948 was responsible for the selection of few tetracycline-resistant clones particularly adapted to the human host, thereby causing the emergence of GBS diseases in neonates in the 60s [7]. GBS express a capsular polysaccharide (CPS) and ten serotypes have been described to date (Ia, Ib, and II–IX). While GBS serotype III strains are strongly associated with neonatal meningitis, serotype V isolates emerged in the United States as the most frequent serotype causing invasive disease in nonpregnant adults, followed by serotypes Ia and III [5]. These three capsular serotypes are also associated with the vast majority of invasive infections in several European countries [8–10]. The only available treatment against GBS infections is based on the use of antibiotics. The implementation of the *intrapartum* antibiotic prophylaxis (IAP) in colonized pregnant women contributed to the decrease of the early-onset manifestations of GBS diseases (infection occurring in the first week of life). However, the IAP treatment has contributed to the emergence of antibiotic-resistant clones [6] with no effect on the late-onset neonatal disease (infections occurring between the first week and the first month of life). The rate of adult GBS disease has not declined until now and the use of antibiotics will likely cause increased resistance as observed with neonatal GBS isolates. Therefore, the development of a vaccine, as an alternative approach to the current use of antibiotics, will benefit neonates, pregnant and nonpregnant adults [11].

GBS vaccines have been initially developed by coupling capsular polysaccharide (CPS) antigens to immunogenic protein carriers but the existence of distinct epitope-specific capsular serotypes has hampered the development of a global GBS vaccine [12]. To avoid the selection of mutants that escape immune recognition, the ideal human GBS vaccine should be directed against structurally conserved antigens that are essential for GBS virulence and/or growth, but none of the hitherto described candidate antigens fulfills these requisites.

We showed in a mouse model of neonatal GBS infection that GBS glyceraldehyde-3-phosphate dehydrogenase (GAPDH) is a valuable vaccine candidate. Maternal vaccination with the recombinant form of this protein was highly effective in protecting the offspring against a lethal infection with GBS [13]. Importantly, the antibodies raised against the bacterial GAPDH did not react with the human GAPDH [13]. However, before being used in clinical practice, a vaccine must pass several rigorous pre-clinical tests to evaluate its safety, effectiveness, or possible side effects [14]. Therefore, in this study, we conducted a comprehensive series of experiments to evaluate the stability, systemic toxicity, and local reactogenicity of the rGAPDH vaccine. These experiments were designed to identify any potential systemic and organ-specific toxicity, and to evaluate the stability of the vaccine formulation. Moreover, given the increased cases of adult infections with this pathogen, we assessed the effectiveness of rGAPDH vaccine against the infection caused by GBS in adult susceptible mice using two GBS strains, A909 and 2603V/R, belonging to serotypes Ia and V, respectively. Since diabetes is present in 20–40% of non-pregnant adults infected with GBS [2, 4, 5], we also tested the efficacy of rGAPDH vaccine in mice rendered diabetic. The obtained results showed that rGAPDH vaccine is highly immunogenic and stable for at least 12 months at 4°C, and no systemic or organ specific toxicity were observed. The protective assays proved that the vaccine constituted by GAPDH is effective against the infections caused by GBS in susceptible and diabetic adult mice. These results identify GBS GAPDH as a valuable global human vaccine to prevent neonatal and adult GBS diseases.

## Materials and Methods

### Animals

Balb/cAnNCrl mice were purchased from Charles River (Italy). All the animals were kept in the animal facilities of the Institute Abel Salazar during the time of the experiments. Mice were 6–8 weeks of age at the beginning of experiences. They were housed in Techniplast ventilated polycarbonate cages under positive pressure with hardwood bedding and provided with Mucedola Diet and fresh tap water, *ad libitum*, throughout the study. All animals were housed in environmentally controlled cages with 40 air changes per hour.

### Ethics statement

This study was carried out in strict accordance with the recommendations of the European Convention for the Protection of Vertebrate Animals used for Experimental and Other Scientific Purposes (ETS 123) and Directive 2010/63/EU and Portuguese rules (DL 113/2013). The animal experimental protocol was approved by the competent national authority Direção Geral de Alimentação e Veterinária (DGAV) (Protocol Permit Number: 0420/000/000/2008). All animal experiments were planned in order to minimize mice suffering.

### Bacteria

GBS A909 (NEM2526) and 2603V/R (NEM2433) belong to capsular serotype Ia and V, respectively. They were grown in Todd-Hewitt broth or agar (Difco Laboratories) at 37°C.

### Purification of recombinant GAPDH

*E*. *coli* BL21 (DE3) strain (Novagen) and the pET28a plasmid (Novagen) were used for production of the recombinant GAPDH (rGAPDH) protein from GBS NEM316 as described previously [15].

### Formulations

rGAPDH vaccine for protection and safety studies were formulated prior to immunization with 10 μg (V10 group), 20 μg (V20 group), or 40 μg (V40 group) of protein, a dose corresponding to 0.5, 1 and 2 mg/kg of rGAPDH, respectively, in a 1:40 PBS-Alhydrogel suspension. The sham-immunized control animals received 200 μL of PBS (Vehicle control without Alhydrogel) or a 1:40 PBS-Alhydrogel suspension (Vehicle control with Alhydrogel). The vaccine used in stability studies was formulated immediately after rGAPDH purification with 20 μg of rGAPDH in a final volume of 200 μL of a 1:40 PBS-Alhydrogel suspension (Aluminium hydroxide Gel; Brenntag) and was maintained at 4°C for 0, 6, and 12 months (S0, S6 and S12 groups, respectively).

### Vaccine safety studies

A total of 30 female Balb/c mice, in groups of 6, were injected s.c. three times, with a three-week intervening period, with 200 μL of V10, V20, or V40. The sham-immunized controls received 200 μL of PBS or 1:40 PBS-Alhydrogel suspension.

Cage side observations were performed daily and included evaluation of mortality, morbidity, general health, and signs of toxicity. Clinical observations were performed twice a week. Body weight was determined twice a week. Reactogenicity of immunization site was scored for edema, ulcer, and erythema using a scale from zero for no symptoms to four for severe symptoms. Reactogenicity scoring was performed on all the mice in each study group after each dosing. One day after administration of the last dose (Day 43), all animals were fasted overnight and euthanized for interim necropsy. Prior to necropsy, terminal blood was collected from all mice under Rompum/Imalgene 1000 anesthesia. Plasma was used for clinical chemistry analyses which included alanine aminotransferase (ALAT), aspartate aminotransferase (ASAT), albumin, amylase, lactate dehydrogenase (LDH), complement component 3 (C3), creatine kinase (CK), creatine kinase MB (CK-MB), total protein, creatinine, total bilirubin, glucose and Clara cell secretory protein 16 (CC16). Moreover, quantitative tests of 24-hour urine were also carried out with metabolic cages, for the evaluation of the levels of creatinine, N-acetyl-beta-D-glucosaminidase (NAG), and urea. All reagents were obtained from PZ Cormay S.A with exception of NAG reagents that were obtained from Diazyme Europe GmbH.

Plasma biochemical parameters were measured in duplicate on an AutoAnalyser (PRESTIGE 24i, PZ Cormay S.A). Urinary urea, creatinine and total proteins were measured in duplicate according to previously described methods [16, 17]. Plasmatic Clara cell 16 was quantified with an Enzyme Linked Immunosorbent Assay kit (USBiological) used according to the manufacturer's instructions.

Animals were subjected to a full gross necropsy. External features suggesting any abnormality, especially evidence of lymph node enlargement was register. After opening the chest and abdominal cavities, an *in situ* examination was done. Heart, lungs, liver, spleen, kidneys, gastrointestinal tract, pancreas, thymus and injection site were studied. The individual organs (liver, spleen, kidney, lung and heart) were removed and re-examined for gross morphology changes. After examination, they were weighed, adequately sliced and fixed in 4% (v/v) neutral buffered paraformaldehyde by diffusion, for 24h, and subsequently dehydrated with graded ethanol and included in paraffin blocks. Xylene was used in the transition between dehydration and impregnation. Sections of 5 μm were cut from paraffin blocks on a microtome (Leica Microsystems, Model RM2125) and mounted on silane coated slides. After dewaxing with xylene and rehydrated with graded alcohol, slides were stained with hematoxylin/eosin and examined under a light microscope (Zeiss Axio ImagerA1) by a certified veterinary pathologist. For every visual field, the histopathological evidences of tissue damage were analyzed as previously described [18] and a total histopathological score was calculated for each organ, allowing the comparison among all groups.

### Vaccine stability studies

A total of 20 female Balb/c mice, in groups of 4, were injected subcutaneously (s.c.), three times, with a three-week intervening period, with 200 μL of S0, S6 and S12 preparations containing 20 μg of rGAPDH in a 1:40 PBS-Alhydrogel suspension. The sham-immunized control animals received 200 μL of PBS or a 1:40 PBS-Alhydrogel suspension. Cage side and clinical observations as well as evaluation of the reactogenicity of immunization site were performed as described above for safety vaccination studies.

### Immunoprotection studies

Balb/c mice were injected subcutaneously (s.c.), three times, with a 3-week intervening period with 20 μg dose of rGAPDH in a 1:40 PBS-Alhydrogel suspension. The sham-immunized control animals received 200 μL of 1:40 PBS-Alhydrogel suspension. Mice were infected i.p. with 0.3 ml of PBS containing 3x10^6^ CFU of GBS A909, or 3x10^7^ CFU of GBS 2603V/R, and sacrificed at indicated timepoints. Survival curves were determined over a 20-day experimental period. Prior to necropsy, terminal blood was collected from all mice under Rompum/Imalgene 1000 anesthesia. Blood was collected and analyzed for GBS counts and the serum was used for cytokine analysis. The analyzed organs were aseptically removed, homogenized in PBS and serial dilutions of homogenized organs were plated on Todd-Hewitt agar to enumerate bacterial CFU.

### Diabetes mouse model

Balb/c mice were injected subcutaneously (s.c.), three times, with a 3-week intervening period with 20 μg dose of rGAPDH in a 1:40 PBS-Alhydrogel suspension. The sham-immunized control animals received 200 μL of 1:40 PBS-Alhydrogel suspension. One week after the second immunization, diabetes was induced by i.p. administration of a single dose of streptozotocin (Sigma) at 200 mg/kg freshly dissolved in 0.05M citrate buffer, pH 4.5 [19]. Plasma glucose levels were measured by OneTouch Verio blood glucose meter system (LifeScan, Johnson and Johnson Company). Serum glucose levels of control animals ranged from 75–195 mg/dl. Mice showing non-fasting serum glucose levels above 600 mg/dl at the time of the third immunization STZ-injection were considered diabetic and used for the study. Mice were infected i.p. with 0.3 ml of PBS containing 3x10^7^ CFU of GBS 2603V/R one week after the last immunization and sacrificed 18h post-infection. Prior to necropsy, terminal blood was collected from all mice under Rompum/Imalgene 1000 anesthesia. Peritoneal lavage was performed with 5 mL of ice-cold PBS. The analyzed organs were aseptically removed, homogenized in PBS and serial dilutions of homogenized organs were plated on Todd-Hewitt agar to enumerate bacterial CFU.

### Quantification cytokines and CRP

TNF-α, IL-6, IL-1β and C-reactive protein (CRP) were quantified with an Enzyme Linked Immunosorbent Assay kit (eBioscience) used according to the manufacturer's instruction.

### Antibody titration

Total IgG and rGAPDH-specific IgG antibodies titers were assessed in the plasma of immunized mice by Enzyme Linked Immunosorbent Assay. Briefly, serial dilutions of the serum of immunized mice were added to the wells of a microtiter plate (NUNC) coated with rGAPDH (5 μg/mL) for two hours at room temperature.

A conjugated goat anti-mouse IgG-HRP (H+ L, 1:1000, SouthernBiotech) antibody was then added and the plate was incubated for two additional hours at room temperature. The *o*-phenylenediamine substrate solution (Pierce) was added and, after addition of the stop solution, the color reaction was measured immediately by the absorption at 450 nm using a spectrophotometer (Thermo Multiskan Ex).

### Statistical analysis

All statistical analyses were performed in GraphPad Prism version 5.0 for Windows (GraphPad Software, San Diego, California). For safety studies, one-way ANOVA with post-hoc Dunnett's Multiple Comparison Test with 95% of confidence was used to analyze the differences between all groups and Alhydrogel group. For stability studies, a one-way ANOVA with post-hoc Tukey’s t-test with 95% of confidence was used to compare the different tested groups. For colonization and cytokine analysis, unpaired Student’s t-test was used to determine the differences between GAPDH-immunized and sham-immunized groups. For survival curve analysis, Mantel-Cox test was performed. Considering the abnormal distribution of the histopathological score data, differences among groups were tested using the nonparametric Kruskal-Wallis test followed by Dunn's test. A P value < 0.05 was considered statistically significant.

## Results

### Safety studies evaluation

#### Mortality and clinical observations

All mice survived to the assigned end point and appeared outwardly healthy after exposure to the vaccine, with no visible physical disability or behavior alterations.

#### Reactogenicity

Small nodules (< 3 mm) were only observable after necropsy at the site of each injection in mice of the groups V10, V20, V40 and Alhydrogel alone. The nodules were too small to be noticed by touch during reactogenicity observations. This was considered as a normal reaction resulting from the vehicle components. Signs of edema or erythema were not observable in any animal during the time of experiment.

#### Body weight and body weight increase

No significant effects on body weight and body weight increase were observed during the study among all test groups. Overall, most animals gained weight throughout the study.

#### Anatomical pathology and histopathology

There were no significant changes in organ weights (Table 1) and no gross morphological changes. Microscopic analysis was performed on several sections from different locations in every organ with a magnification of 40x in order to guarantee a global and precise organ overview. This examination took into account the severity of tissue organization, the degree of cellular degeneration, the amount of interstitial inflammatory cells, and the existence and extension of tissue necrotic areas. In all groups, none of these histopathological traits was detected in the vital organs studied and the total histopathology score calculated for each organ did not show significant differences among groups (data not shown).

**Table 1:** Organ weight in mice with different vaccine doses.

Organ	Vehicle control (w/out Alhydrogel)	Vehicle control (w/ Alhydrogel)	rGAPDH (with Alhydrogel)		
			V10	V20	V40
**Heart**	0.006 ± 0.001	0.006 ± 0.001	0.006 ± 0.001	0.006 ± 0.001	0.006 ± 0.001
**Lungs**	0.52 ± 0.04	0.50 ± 0.04	0.59 ± 0.11	0.58 ± 0.09	0.51 ± 0.05
**Kidneys**	2.02 ± 0.33	2.14 ± 0.29	2.11 ± 0.34	2.40 ± 0.39	2.19 ± 0.20
**Liver**	8.91 ± 1.41	9.11 ± 1.34	8.99 ± 1.31	7.84 ± 1.42	9.74 ± 0.92
**Spleen**	0.08 ± 0.01	0.07 ± 0.01	0.08 ± 0.01	0.08 ± 0.01	0.08 ± 0.01

Values are expressed as mean ± S.D; Number of animals per goup = 6; V10—10 μg s.c.; V20—20 μg s.c.: V40—40 μg s.c. One-way ANOVA with post-hoc Dunnett's Multiple Comparison Test, p > 0.05.

### Immunogenicity

The efficacy of a vaccine is closely associated with the strength of the induced immune response [14]. To confirm the immunogenicity of vaccine, titers of IgG specific for rGAPDH were determined in the serum of the animals twenty-four hours after the last immunization.

**Fig 1:**
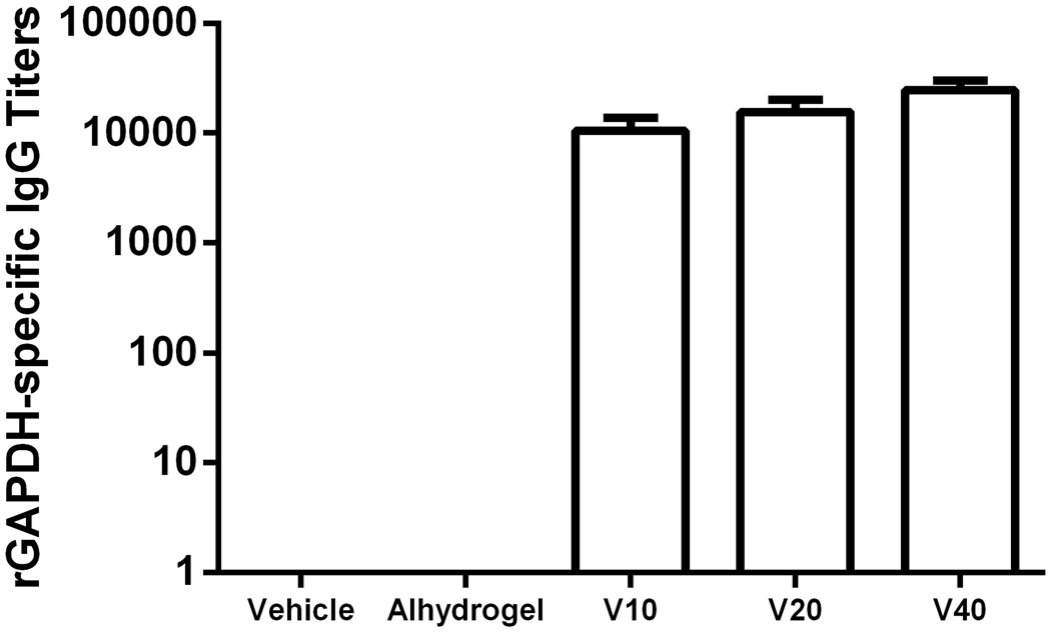
rGAPDH vaccine is immunogenic at all tested doses. rGAPDH-specific IgG titers of 6 female Balb/c mice per group, immunized three times with a three week interval, 24h after the third immunization with 10 (V10), 20 (V20) or 40 (V40) μg of rGAPDH with 1:40 Alhydrogel, alhygrogel alone, or PBS. The ELISA plates were coated with rGAPDH and revealed with goat anti-mouse IgG—HRP. Titers are represented as minimal serum dilution necessary for the lost absorbance signal.

All tested vaccine doses (V10, V20, and V40) induced the production of IgG antibodies against rGAPDH (Fig 1). The titer values show a tendency to increase with the dose of rGAPDH-Alhydrogel injected, but the observed differences are not statistically significant. As expected, rGAPDH-specific IgG antibodies were not detected in the serum of the controls (Vehicle and Alhydrogel).

### Plasma and urine chemistry

The serological and urine biochemical parameters were evaluated in all animals and found to be in normal range after repeated exposure to the vaccine compounds (Table 2). The parameters evaluated were: hepatotoxicity by quantifying the levels of alanine transaminase (ALT), aspartate transaminase (AST), lactate dehydrogenase (LDH) and albumin [20]; cardiotoxicity by assessing the levels of creatine kinases (CK) and specifically its isoform creatine kinase-MB (CK-MB) [21]; and nephrotoxicity by measuring the levels of urea, creatinine and N-acetyl-beta-D-glucosaminidase [22]. Moreover, to evaluate pancreatic and lung toxicity, the amylase and clara-cell 16 were analyzed, respectively [23, 24]. The systemic toxicity was also considered by quantifying the serum levels of glucose and total proteins.

**Table 2:** Biochemistry of plasma and urine collected 24h after the last vaccine injection.

	Vehicle control (w/out Alhydrogel)	Vehicle control (w/ Aldhydrogel)	rGAPDH (with Alhydrogel)		
			V10	V20	V40
**Plasma Parameters**					
**Albumin (g/L)**	32.61 ± 2.21	29.19 ± 4.65	29.86 ± 1.19	31.89 ± 1.81	30.91 ± 2.22
**Total proteins (g/L)**	51.84 ± 3.41	48.04 ± 1.09	47.22 ± 1.96	48.98 ± 3.08	47.34 ± 2.24
**Glucose (mg/dL)**	85.7 ± 30.7	106.6 ± 29.9	90.5 ± 25.8	86.2 ± 10.8	87.0 ± 25.8
**CK-MB (U/L)**	227.9 ± 30.3	230.9 ± 38.8	236.0 ± 97.6	224.1 ± 67.9	196.2 ± 53.8
**CK (U/L)**	1111 ± 265	1192 ± 397	1754 ± 721	1460 ± 476	1175 ± 546
**ALAT (U/L)**	31.7 ± 2.9	35.1 ± 11.5	34.0 ± 2.9	29.4 ± 4.7	31.0 ± 3.5
**ASAT (U/L)**	99.0 ± 19.2	92.5 ± 20.7	111.1 ± 37.2	112.1 ± 33.4	90.2 ± 26.7
**LDH (mg/dL)**	743.6 ± 360.7	633.6 ± 234.7	625.6 ± 107.7	677.4 ± 65.6	623.9 ± 203.5
**Amylase (U/L)**	966.2 ± 262.2	870.5 ± 197.6	1000 ± 251.9	867.2 ± 167.9	806.8 ± 101.9
**C3 (mg/mL)**	11 ± 3	11 ± 3	14 ± 4	9 ± 3	14 ± 5
**Urea (mg/dL)**	46.3 ± 11.3	47.9 ± 10.3	53.2 ± 22.6	45.5 ± 5.8	45.3 ± 9.7
**Creatinine (mg/dL)**	0.35 ± 0.08	0.35 ± 0.05	0.28 ± 0.12	0.35 ± 0.05	0.30 ± 0.10
**CC16 (ng/mL)**	125.1 ± 43.1	154.9 ± 29.3	161.3 ± 37.9	121.2 ± 55.5	116.4 ± 25.6
**Urine Parameters**					
**Urea (mg/24h)**	4.47 ± 3.40	5.18 ± 2.68	5.48 ± 3.68	4.46 ± 2.35	4.91 ± 2.41
**Creatinine (mg/dL)**	57 ± 24	65 ± 14	70 ± 18	67 ± 22	66 ± 25
**NAG (U/L)**	12.04 ± 2.63	11.71 ± 3.95	15.80 ± 1.95	14.57 ± 3.6	11.56 ± 3.5
**Inflammatory Parameters**					
**IL-1β (pg/mL)**	BDL	BDL	BDL	BDL	BDL
**IL-6 (pg/mL)**	BDL	BDL	BDL	BDL	BDL
**TNF-α (pg/mL)**	BDL	BDL	BDL	BDL	BDL
**CRP (ng/mL)**	2.30 ± 0,76	2.50 ± 0.81	2.30 ± 0.30	2.40 ± 0.31	2.23 ± 0.62

Values are expressed as mean ± S.D; Number of animals per group = 6; BDL—Below detection Level; CK—Creatine kinase; CK-MB—Creatine kinase MB; ALAT—Alanine aminotransferase; ASAT—Aspartate aminotransferase; LDH—Lactate dehydrogenase; C3—Complement component 3; CC16—Clara cell secretory protein 16; NAG—N-acetyl-beta-D-glucosaminidase; IL—Interleukine; TNF-α—Tumor necrosis factor α; CRP—C-reactive protein. One-way ANOVA with post-hoc Dunnett's Multiple Comparison Test, p > 0.05.

### Inflammatory parameters

Acute-phase proteins were used as markers for acute inflammation induced after rGAPDH immunization. These acute-phase proteins are produced by cells or tissues in response to inflammatory stimulus. The degree of inflammation can be evaluated by measuring the serum level of these proteins [25].

The levels of the pro-inflammatory cytokines IL-1β, TNF-α, and IL-6 in the serum of the animals were below detection level in all groups and the levels of C-reactive protein were not significantly different between groups (Table 2).

### Stability studies evaluation

The shelf-life is an important feature for any vaccine or drug to be used in humans, especially when intended for use in low-income countries where it is often difficult to provide appropriate storage conditions [26]. The vaccine formulations were maintained at 4°C for 6 (S6) and 12 (S12) months before immunization. An immunization protocol similar to that of the safety studies was used and the potency of the formulations was evaluated by comparing the resulting specific antibody titers with those obtained with a fresh prepared formulation (S0).

### Antibody titers

**Fig 2:**
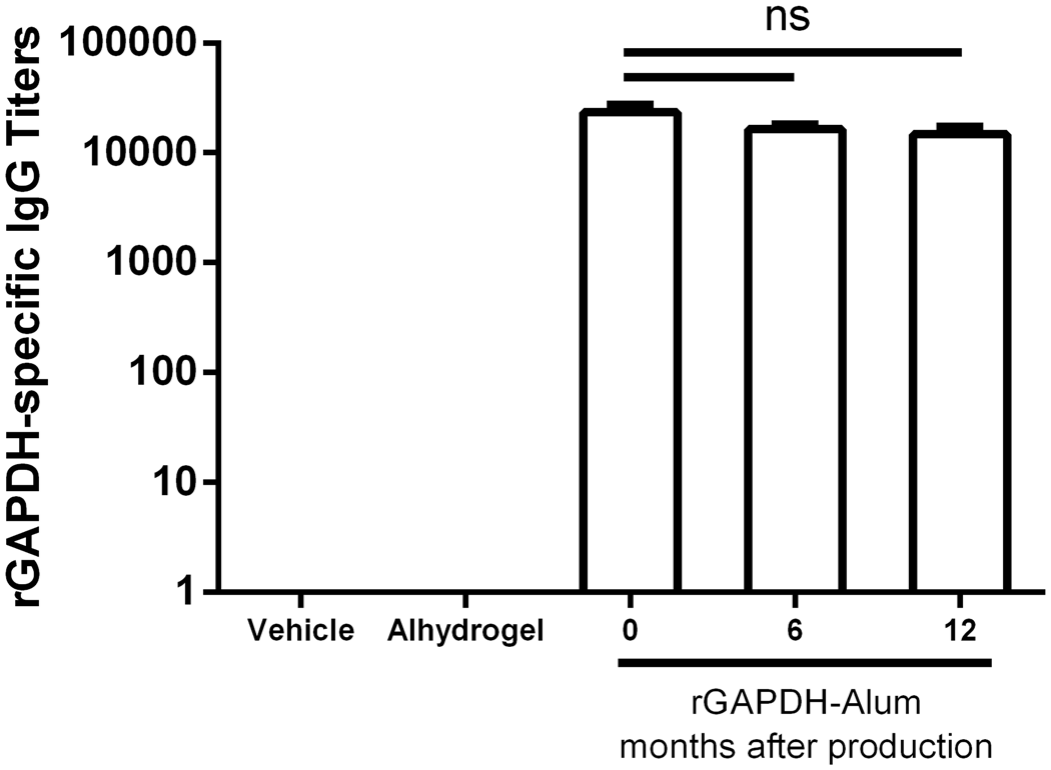
rGAPDH vaccine preserves its potency after prolonged storage at 4°C. rGAPDH-specific IgG titers of 4 female Balb/c mice per group, immunized three times with a three week interval, 24 h after the third immunization with PBS, Alhydrogel and 20 μg of rGAPDH in a 1:40 PBS/Alhydrogel suspension prepared fresh (S0) or conserved at 4°C for 6 (S6) and 12 (S12) months. The ELISA plates were coated with rGAPDH and revealed with goat anti-mouse IgG—HRP. Titers are represented as minimal serum dilution necessary for the lost absorbance signal. One-way ANOVA with post-hoc Tukey’s t-test. ns—not significant.

The titers of IgG antibodies against rGAPDH induced by vaccines stored at 4°C for 6 and 12 months were similar to those induced by a freshly prepared vaccine (Fig 2). Moreover, as expected, rGAPDH-specific IgG antibodies were not detected in the serum of the controls (Vehicle and Alhydrogel) (Fig 2). The levels of total IgG were similar between all groups (vaccinated and controls, data not shown). The storage of the vaccine did not alter the potency or the specificity of the response.

### Safety assessment of S0, S6, and S12 formulations

Treatment with S6 and S12 formulations had no life-threatening effect. All mice survived to the assigned end point and appeared healthy outwardly after exposure to the vaccine, with no visible physical disability or behavior alterations, and with no difference in body weight associated with the immunization. The treatment with the vaccines and adjuvant alone produced small nodules only observable after necropsy at the site of injection. The levels of the pro-inflammatory cytokines IL-1β, TNF-α, and IL-6 in the serum of the animals were below detection threshold in all groups. The levels of C-reactive protein were not significantly different between groups (Data not shown).

### rGAPDH vaccination protects susceptible and diabetic adult mice from GBS infections

**Fig 3:**
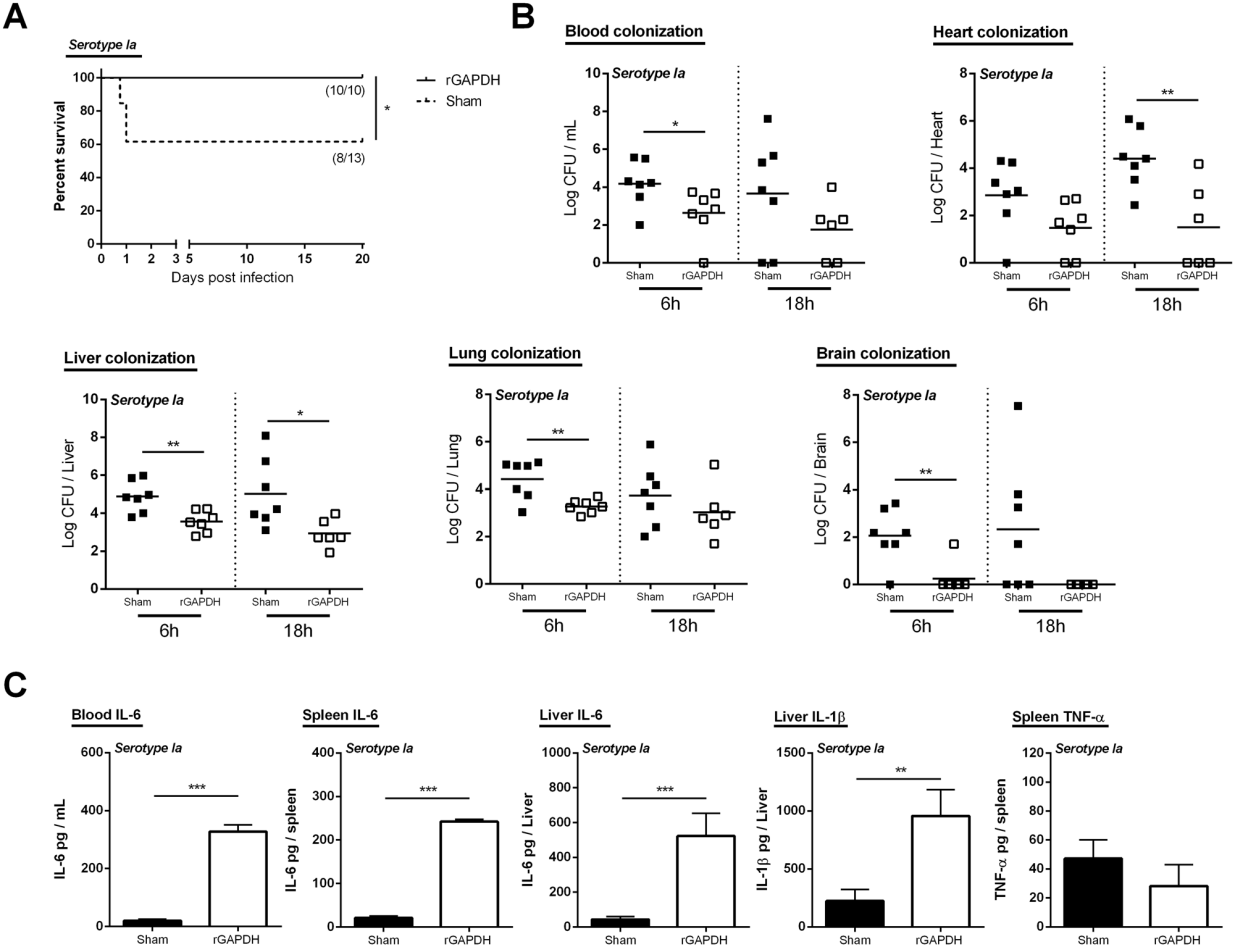
rGAPDH vaccination improves survival and induces protection against serotype Ia GBS infection in an adult mouse model. A) Kaplan–Meier survival curves. The lethality was monitored for 20 days. The numbers in parentheses represent the number of animals that survived out of the total number of infected animals. B) Blood, liver, lung, heart and brain colonization 6 and 18 hours post-infection (6 hours—Sham and GAPDH-immunized n = 7; 18 hours—Sham n = 7, rGAPDH-immunized n = 6) and C) sera, liver and spleen cytokine production 3 hours post-infection (n = 6 for both groups). Balb/c mice were immunized three times, with a three week interval, with 20 μg of rGAPDH in 1:40 Alhydrogel or treated with Alhydrogel alone and infected i.p. with 3x10^6^ CFU of A909 (serotype Ia). Unpaired Student’s t-test. *p < 0.05; **p < 0.01; *** p < 0.001.

**Fig 4:**
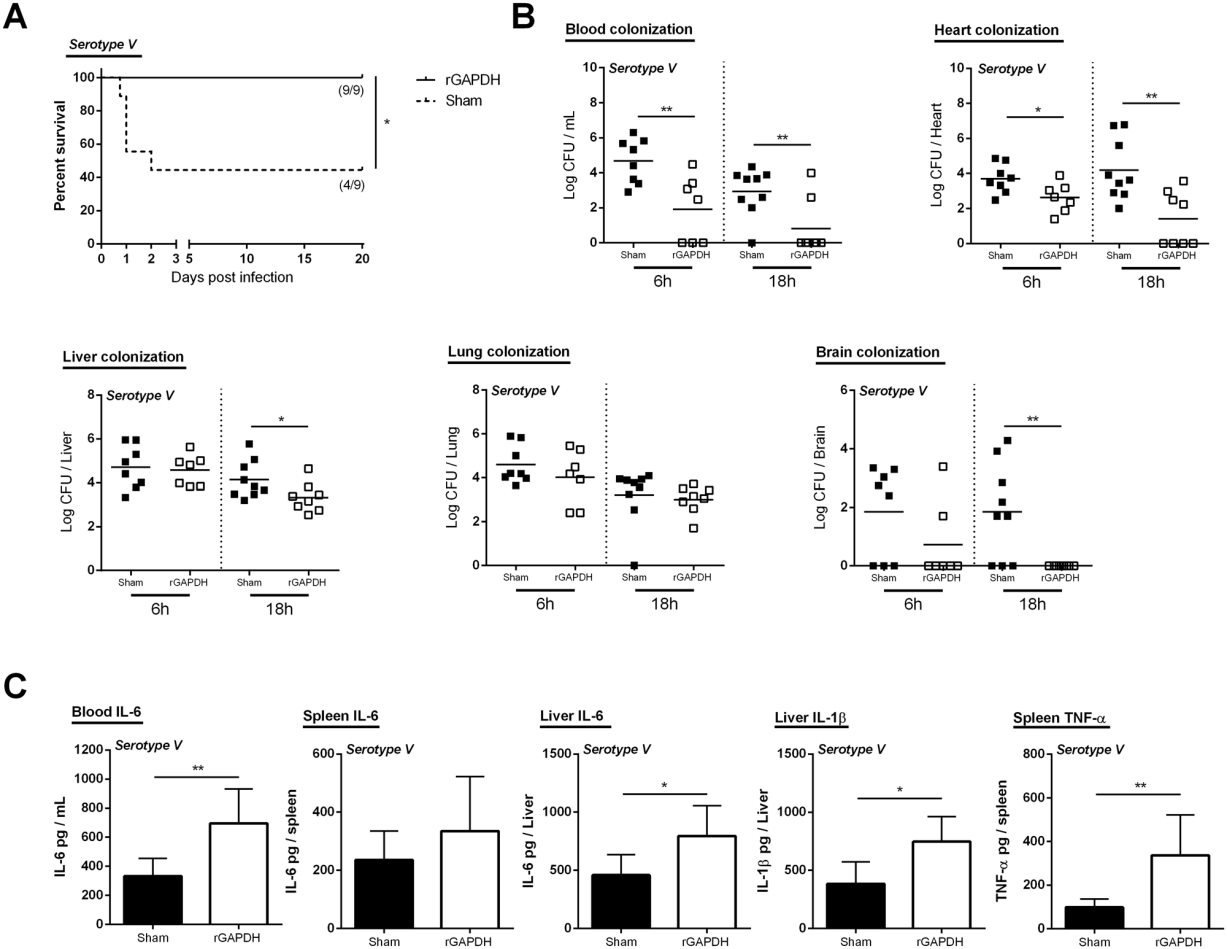
rGAPDH vaccination improves survival and induces protection against serotype V GBS infection in an adult mouse model. A) Kaplan–Meier survival curves. The lethality was monitored for 20 days. The numbers in parentheses represent the number of animals that survived out of the total number of infected animals. B) Blood. liver. lung. heart and brain colonization 6 and 18 hours post-infection and (6 hours—Sham n = 8. GAPDH-immunized n = 7; 18 hours—Sham n = 9. rGAPDH-immunized n = 8) C) sera. liver and spleen cytokine production 3 hours post-infection (n = 6 for both groups). Balb/c mice were immunized three times, with a three week interval, with 20 μg of rGAPDH in 1:40 Alhydrogel or treated with Alhydrogel alone and infected i.p. with 3x10^7^ CFU of 2603V/R (serotype V). Unpaired Student’s t-test. *p < 0.05; **p < 0.01; *** p < 0.001.

To assess the effectiveness of rGAPDH vaccine against GBS infections in adult mice, immunized adult Balb/c mice were infected with A909 (serotype Ia) or 2603V/R (serotype V). As shown in Figs 3 and 4, rGAPDH immunization confers protection to adult mice against GBS infections caused by both serotypes. The survival of rGAPDH-vaccinated mice, either infected with serotype Ia or V, were significantly increased compared with the respective sham-immunized group (Figs 3 and 4). Indeed, none of the immunized mice succumbed to the infection (100% survival) whereas ~40% and ~60% of sham-immunized mice infected with serotype Ia and V, respectively, died within the first 48h (Figs 3A and 4A). To investigate whether the longer survival of rGAPDH-vaccinated mice was associated with an early control of GBS growth, we next determined organ colonization at 6h and 18h post-infection. When infected with GBS serotypes Ia or V, lower numbers of viable bacteria were found in blood, heart, liver, and brain of the rGAPDH-immunized mice, as compared with the sham-immunized controls (Figs 3B and 4B). Lower bacterial levels were also found in the lung of rGAPDH-immunized animals infected with serotype Ia (Fig 3B). Since non-pregnant adult humans with diabetes are highly susceptible to GBS infections, we also evaluated the efficacy o rGAPDH vaccine in mice rendered diabetic by streptozotocin induction and infected with GBS 2603V/R, i.e. a strain belonging to the emerging serotype causing invasive disease in non-pregnant adults. As shown in Table 3, rGAPDH vaccination rendered the diabetic mice more resistant to GBS infection, compared with sham-vaccinated diabetic mice. Indeed, 18h post-infection, lower levels of GBS CFUs were observed in blood, heart, liver, peritoneum, kidney, spleen and brain of the rGAPDH-immunized compared with the sham-immunized controls (Table 3).

**Table 3:** Organ colonization of rGAPDH and Sham-immunized diabetic mice infected with GBS serotype V strain 2603V/R.

	Diabetic mice		
Organs	Sham-immunized (log CFU/organ)	rGAPDH-immunized (log CFU/organ)	P-value^a^
Blood	4.35 ± 0.732	0.902 ± 0.462	0.0010***
Liver	5.22 ± 0.550	3.06 ± 0.216	0.0017**
Spleen	4.76 ± 0.505	3.16 ± 0.460	0.0329*
Lung	4.60 ± 0.665	2.46 ± 0.501	0.0198*
Brain	2.61 ± 0.625	0.189 ± 0.189	0.0014**
Heart	4.43 ± 0.908	1.52 ± 0.511	0.0114*
Kidney	4.18 ± 0.754	1.92 ± 0.300	0.0107*
Peritoneum	5.69 ± 0.887	2.93 ± 0.621	0.0204*

Mice were killed 18h post-infection and the organs collected for bacterial counts. Values are expressed as mean ± S.E.M.; Number of animals Sham-immunized = 8. rGAPDH-immunized = 9;

^a^Student’s t Test.

*p<0.05;

**p<0.01.

***p<0.001

To characterize the pro-cytokines associated with protection in rGAPDH-immunized mice, IL-6, TNF-α, and IL-1β were analyzed 3h post-infection in blood, liver and spleen of both groups. As shown in Figs 3C and 4C, an increased production of the pro-inflammatory cytokines, mainly IL-6 and IL-1β, were observed in vaccinated mice. Indeed, compared to the sham-immunized controls, the rGAPDH-immunized mice infected with either GBS serotype Ia or V, presented higher levels of IL-6 in sera, spleen, and liver, and of IL-1β in liver. Higher levels of TNF-α were observed in the spleen of immunized mice infected with GBS serotype V (Fig 4C).

## Discussion

In the past few decades GBS infections have been responsible for a significantly high morbidity and mortality rates amongst nonpregnant adults, particularly in elderly or individuals with chronic underlying conditions, such as diabetes [2–5]. Currently, the invasive disease in adults is increasing and represents about two-thirds of all cases of GBS infections, with a case fatality ratio of about 10–20% [4]. The antibiotic use as prophylactic measure in pregnancy and as treatment in adult against GBS infections raised several obvious concerns regarding the emergence and dissemination of antibiotic-resistant clones [6, 7, 27]. Moreover, while the use of IAP for prevention of GBS disease since 1996 was highly effective at preventing early-onset diseases, it did not changed the rate of late onset diseases, as well as the rates of stillbirths and prematurity caused by GBS infections [28]. Thus, vaccination is considered as a promising and complementary alternative since it could be easily and broadly applicable and could protect not only adults, but also newborns without the inherent limitations and problems associated with antibiotic use [29].

Since low levels of maternal antibodies to capsular polysaccharide antigens correlate with neonatal susceptibility, efforts were concentrated in the production of a vaccine based on the GBS capsular polysaccharide [30, 31]. However, this strategy is not consistent with the fact that the distribution of the ten GBS serotypes can vary in space and time [32, 33]. Moreover, capsular polysaccharide switching has been recently reported in GBS [34]. The possibility of a shift in serotype prevalence supports the need for a universal GBS vaccine based on a different antigen [35]. Accordingly, Grandi and colleagues developed a vaccine utilising a component of each of the three pilus identified so far in GBS [36]. However, a change in the pili variant was described to occur in the case of *Neisseria gonorrhoeae*, rendering a promising vaccine ineffective [37]. Based on this observation, one might predict that, following vaccination with pilus antigen, the immune selective pressure will select GBS pilus variants to escape immunity.

We have identified GAPDH as a valuable universal GBS vaccine candidate [13, 15]. In a mouse model of GBS infection, maternal vaccination with rGAPDH or passive immunization with anti-rGAPDH IgG antibodies conferred neonatal protection [13]. Since GAPDH is ubiquitously expressed in all type of cells, including mammalian cells, we demonstrated that the antibodies produced against the GBS GAPDH do not react with its human counterpart [13]. Eukaryotic and prokaryotic GADPH sequences display only two 10-aminoacid long identical segments and we consistently showed that antibodies produced against either native or denatured GBS GAPDH do not recognize human GAPDH [13].

In the present work, the protective effect of this vaccine was extended to adult susceptible mice. We showed that adult mice vaccinated with rGAPDH are protected against GBS infections caused by the two serotypes, Ia and V, mostly associated with the human invasive disease [5]. The survival of rGAPDH-vaccinated adult mice is significantly increased compared with sham-vaccinated animals. Mice vaccinated with rGAPDH presented decreased number of bacterial counts in blood, brain, and heart, compared with sham-immunized animals. This observation is particularly important since endocarditis and meningitis are the two clinical presentations of GBS invasive infections with the worst prognosis in terms of morbidity and mortality [38, 39]. The rising incidence rate of GBS infections in non-pregnant adults has been associated with the aging of the population and with the increasing prevalence of individuals with underlying comorbidities, like diabetes mellitus [4, 5]. Thus, to mimic the impact of diabetes in the susceptibility to GBS infections, we determined the efficacy of the rGAPDH vaccine in a mouse model of streptozotocin-induced diabetes. Our results show that vaccination with rGAPDH protects adult diabetic mice against GBS serotype V infection.

GAPDH is an essential cytoplasmic enzyme involved in the glycolytic pathway which, despite the lack of standard signal sequences, has been found at the surface of unrelated GBS isolates [13]. This abundant enzyme is most likely released upon cell lysis to then bind to the surface of living bacteria [40]. GAPDH possesses a critical metabolic function, being essential for bacterial growth in blood, and plays an important role in GBS virulence. Alike GAPDH, other metabolic and cytosolic proteins have been also detected at the surface of numerous microorganisms where they exert a distinct function, being therefore called “moonlighting” proteins [41]. In pathogens, extracellular “moonlighting” proteins are often involved in colonization and invasion of host tissues and we previously reported that cell surface bound GAPDH confers to GBS the ability to bind plasminogen and fibrinogen [42, 43] and displays immunomodulatory properties that contributes evasion from the host immune system [13, 15]. The potential of these “moonlighting” proteins as a vaccine target have also be experimentally assayed [44–47] in other streptococci. For example, the α-enolase of *Streptococcus sobrinus* and *Streptococcus suis* [46, 47], the fructose-bisphosphate aldolase *of Streptococcus pneumoniae’* [45] and the arginine deiminase and the trigger factor of *Streptococcus pyogenes* [44] were characterized as protective antigens.

To move a candidate vaccine from the laboratory to the clinic, preclinical tests including safety studies in animals are mandatory [14, 48]. The evaluation of toxicological parameters of repeated administration of rGAPDH-Alhydrogel formulations showed no effect on mortality, clinical appearance, behavior, or body weight change. In the first months of life, newborns protection against infectious diseases is highly dependent on passive immunity mediated by mother’s specific IgG antibodies that are transferred trough the placenta [49]. Therefore, a maternal vaccine aimed at protecting the newborn should elicit high levels of IgG antibody [48]. Consistently, our results showed that all tested doses of GAPDH were immunogenic and induced high and similar levels of specific rGAPDH IgG antibodies. Gross and histopathological examinations revealed no obvious abnormalities in organs and tissues of vaccinated mice. Moreover, the biochemical characterization of serum samples confirmed the innocuity of the vaccine deduced from the histological data. The serum levels of acute phase proteins or organ specific markers were not altered by the immunization, which indicates that rGAPDH vaccination does not induce acute inflammation or organ toxicity. Therefore, our study demonstrates the safety and immunogenicity of the rGAPDH vaccine. The WHO guidelines for nonclinical and clinical evaluation of vaccines stress a need for stability data to support clinical trial approval [50] and we showed here that the rGAPDH vaccine conserves its potency and safety, without significant alteration, at 4°C for at least 12 months.

This study predicts a potential application of rGAPDH vaccine in humans to prevent GBS-induced adult and neonatal diseases.

## References

[1] Johri AK, Paoletti LC, Glaser P, Dua M, Sharma PK, Grandi G, et al Group B Streptococcus: global incidence and vaccine development. Nat Rev Microbiol. 2006;4(12):932–42. Epub 2006/11/08. 10.1038/nrmicro1552 17088932 PMC2742968

[2] Blancas D, Santin M, Olmo M, Alcaide F, Carratala J, Gudiol F. Group B streptococcal disease in nonpregnant adults: incidence, clinical characteristics, and outcome. European journal of clinical microbiology & infectious diseases: official publication of the European Society of Clinical Microbiology. 2004;23(3):168–73. 10.1007/s10096-003-1098-9 . 14986167

[3] Jackson LA, Hilsdon R, Farley MM, Harrison LH, Reingold AL, Plikaytis BD, et al Risk factors for group B streptococcal disease in adults. Annals of internal medicine. 1995;123(6):415–20. . 7639440 10.7326/0003-4819-123-6-199509150-00003

[4] Farley MM. Group B streptococcal disease in nonpregnant adults. Clinical infectious diseases: an official publication of the Infectious Diseases Society of America. 2001;33(4):556–61. 10.1086/322696 . 11462195

[5] Skoff TH, Farley MM, Petit S, Craig AS, Schaffner W, Gershman K, et al Increasing burden of invasive group B streptococcal disease in nonpregnant adults, 1990–2007. Clinical infectious diseases: an official publication of the Infectious Diseases Society of America. 2009;49(1):85–92. 10.1086/599369 . 19480572

[6] Schrag SJ, Zywicki S, Farley MM, Reingold AL, Harrison LH, Lefkowitz LB, et al Group B streptococcal disease in the era of intrapartum antibiotic prophylaxis. The New England journal of medicine. 2000;342(1):15–20. 10.1056/NEJM200001063420103 . 10620644

[7] Da Cunha V, Davies MR, Douarre PE, Rosinski-Chupin I, Margarit I, Spinali S, et al Streptococcus agalactiae clones infecting humans were selected and fixed through the extensive use of tetracycline. Nature communications. 2014;5:4544 10.1038/ncomms5544 25088811 PMC4538795

[8] Bergseng H, Rygg M, Bevanger L, Bergh K. Invasive group B streptococcus (GBS) disease in Norway 1996–2006. European journal of clinical microbiology & infectious diseases: official publication of the European Society of Clinical Microbiology. 2008;27(12):1193–9. 10.1007/s10096-008-0565-8 . 18560908

[9] Martins ER, Melo-Cristino J, Ramirez M, Portuguese Group for the Study of Streptococcal I. Dominance of serotype Ia among group B Streptococci causing invasive infections in nonpregnant adults in Portugal. Journal of clinical microbiology. 2012;50(4):1219–27. 10.1128/JCM.05488-11 22219307 PMC3318525

[10] Tazi A, Morand PC, Reglier-Poupet H, Dmytruk N, Billoet A, Antona D, et al Invasive group B streptococcal infections in adults, France (2007–2010). Clinical microbiology and infection: the official publication of the European Society of Clinical Microbiology and Infectious Diseases. 2011;17(10):1587–9. 10.1111/j.1469-0691.2011.03628.x . 21883671

[11] Edwards MS. Group B streptococcal conjugate vaccine: a timely concept for which the time has come. Human vaccines. 2008;4(6):444–8. . 18728401 10.4161/hv.4.6.6507

[12] Johri AK, Paoletti LC, Glaser P, Dua M, Sharma PK, Grandi G, et al Group B Streptococcus: global incidence and vaccine development. Nature reviews Microbiology. 2006;4(12):932–42. 10.1038/nrmicro1552 17088932 PMC2742968

[13] Madureira P, Andrade EB, Gama B, Oliveira L, Moreira S, Ribeiro A, et al Inhibition of IL-10 production by maternal antibodies against Group B Streptococcus GAPDH confers immunity to offspring by favoring neutrophil recruitment. PLoS pathogens. 2011;7(11):e1002363 10.1371/journal.ppat.1002363 22114550 PMC3219712

[14] Forster R. Study designs for the nonclinical safety testing of new vaccine products. J Pharmacol Toxicol Methods. 2012;66(1):1–7. Epub 2012/05/09. S1056-8719(12)00043-3 [pii] 10.1016/j.vascn.2012.04.003 . 22561062

[15] Madureira P, Baptista M, Vieira M, Magalhaes V, Camelo A, Oliveira L, et al Streptococcus agalactiae GAPDH is a virulence-associated immunomodulatory protein. Journal of immunology. 2007;178(3):1379–87. . 17237385 10.4049/jimmunol.178.3.1379

[16] Talke H, Schubert GE. [Enzymatic Urea Determination in the Blood and Serum in the Warburg Optical Test]. Klinische Wochenschrift. 1965;43:174–5. . 14258517 10.1007/BF01484513

[17] Fabiny DL, Ertingshausen G. Automated reaction-rate method for determination of serum creatinine with the CentrifiChem. Clinical chemistry. 1971;17(8):696–700. . 5562281

[18] Dinis-Oliveira RJ, Sousa C, Remiao F, Duarte JA, Navarro AS, Bastos ML, et al Full survival of paraquat-exposed rats after treatment with sodium salicylate. Free radical biology & medicine. 2007;42(7):1017–28. 10.1016/j.freeradbiomed.2006.12.031 . 17349929

[19] Hayashi K, Kojima R, Ito M. Strain differences in the diabetogenic activity of streptozotocin in mice. Biological & pharmaceutical bulletin. 2006;29(6):1110–9. . 16755002 10.1248/bpb.29.1110

[20] Kim SB, Yang WS, Lee SK, Chi HS, Park JS. Effect of increasing serum albumin on haemostatic factors synthesized in the liver in CAPD patients. Nephrology, dialysis, transplantation: official publication of the European Dialysis and Transplant Association—European Renal Association. 1998;13(8):2053–8. . 9719164 10.1093/ndt/13.8.2053

[21] Bozbas H, Yildirir A, Muderrisoglu H. Cardiac enzymes, renal failure and renal transplantation. Clin Med Res. 2006;4(1):79–84. Epub 2006/04/06. 4/1/79 [pii]. 16595795 10.3121/cmr.4.1.79 PMC1435661

[22] Obianime AW, Roberts II. Antioxidants, cadmium-induced toxicity, serum biochemical and the histological abnormalities of the kidney and testes of the male Wistar rats. Nigerian journal of physiological sciences: official publication of the Physiological Society of Nigeria. 2009;24(2):177–85. . 20234761 10.4314/njps.v24i2.52910

[23] Matull WR, Pereira SP, O'Donohue JW. Biochemical markers of acute pancreatitis. Journal of clinical pathology. 2006;59(4):340–4. 10.1136/jcp.2002.002923 16567468 PMC1860356

[24] Bernard A, Lauwerys R. Low-molecular-weight proteins as markers of organ toxicity with special reference to Clara cell protein. Toxicology letters. 1995;77(1–3):145–51. . 7618128 10.1016/0378-4274(95)03284-3

[25] Watterson C, Lanevschi A, Horner J, Louden C. A comparative analysis of acute-phase proteins as inflammatory biomarkers in preclinical toxicology studies: implications for preclinical to clinical translation. Toxicol Pathol. 2009;37(1):28–33. Epub 2009/01/28. 10.1177/0192623308329286 . 19171926

[26] Chen D, Zehrung D. Desirable attributes of vaccines for deployment in low-resource settings. Journal of pharmaceutical sciences. 2013;102(1):29–33. 10.1002/jps.23352 . 23136115

[27] Zeissig S, Blumberg RS. Life at the beginning: perturbation of the microbiota by antibiotics in early life and its role in health and disease. Nature immunology. 2014;15(4):307–10. 10.1038/ni.2847 . 24646587

[28] Jordan HT, Farley MM, Craig A, Mohle-Boetani J, Harrison LH, Petit S, et al Revisiting the need for vaccine prevention of late-onset neonatal group B streptococcal disease: a multistate, population-based analysis. The Pediatric infectious disease journal. 2008;27(12):1057–64. 10.1097/INF.0b013e318180b3b9 . 18989238

[29] Black S, Margarit I, Rappuoli R. Preventing newborn infection with maternal immunization. Science translational medicine. 2013;5(195):195ps11 10.1126/scitranslmed.3005451 . 23884465

[30] Baker CJ, Kasper DL. Group B streptococcal vaccines. Reviews of infectious diseases. 1985;7(4):458–67. . 3898306 10.1093/clinids/7.4.458

[31] Baker CJ, Kasper DL. Correlation of maternal antibody deficiency with susceptibility to neonatal group B streptococcal infection. The New England journal of medicine. 1976;294(14):753–6. 10.1056/NEJM197604012941404 . 768760

[32] Lachenauer CS, Kasper DL, Shimada J, Ichiman Y, Ohtsuka H, Kaku M, et al Serotypes VI and VIII predominate among group B streptococci isolated from pregnant Japanese women. The Journal of infectious diseases. 1999;179(4):1030–3. 10.1086/314666 . 10068604

[33] Persson E, Berg S, Trollfors B, Larsson P, Ek E, Backhaus E, et al Serotypes and clinical manifestations of invasive group B streptococcal infections in western Sweden 1998–2001. Clinical microbiology and infection: the official publication of the European Society of Clinical Microbiology and Infectious Diseases. 2004;10(9):791–6. 10.1111/j.1469-0691.2004.00931.x . 15355409

[34] Bellais S, Six A, Fouet A, Longo M, Dmytruk N, Glaser P, et al Capsular switching in group B Streptococcus CC17 hypervirulent clone: a future challenge for polysaccharide vaccine development. The Journal of infectious diseases. 2012;206(11):1745–52. 10.1093/infdis/jis605 . 23002446

[35] Palmeiro JK, De Carvalho NS, Botelho AC, Fracalanzza SE, Madeira HM, Dalla-Costa LM. Maternal group B streptococcal immunization: capsular polysaccharide (CPS)-based vaccines and their implications on prevention. Vaccine. 2011;29(21):3729–30. 10.1016/j.vaccine.2011.02.102 . 21414381

[36] Margarit I, Rinaudo CD, Galeotti CL, Maione D, Ghezzo C, Buttazzoni E, et al Preventing bacterial infections with pilus-based vaccines: the group B streptococcus paradigm. The Journal of infectious diseases. 2009;199(1):108–15. 10.1086/595564 . 19086816

[37] Hill SA, Davies JK. Pilin gene variation in Neisseria gonorrhoeae: reassessing the old paradigms. FEMS microbiology reviews. 2009;33(3):521–30. 19396954 10.1111/j.1574-6976.2009.00171.x PMC2753986

[38] Domingo P, Barquet N, Alvarez M, Coll P, Nava J, Garau J. Group B streptococcal meningitis in adults: report of twelve cases and review. Clinical infectious diseases: an official publication of the Infectious Diseases Society of America. 1997;25(5):1180–7. . 9402379 10.1086/516094

[39] Sambola A, Miro JM, Tornos MP, Almirante B, Moreno-Torrico A, Gurgui M, et al Streptococcus agalactiae infective endocarditis: analysis of 30 cases and review of the literature, 1962–1998. Clinical infectious diseases: an official publication of the Infectious Diseases Society of America. 2002;34(12):1576–84. 10.1086/340538 . 12032892

[40] Oliveira L, Madureira P, Andrade E, Bouaboud A, Morello E, Ferreira P, et al Group B streptococcus GAPDH is released upon cell lysis, associates with bacterial surface, and induces apoptosis in murine macrophages. PloS one. 2012;7(1). 10.1371/journal.pone.0029963 PMC3264557 22291899

[41] Jeffery CJ. Moonlighting proteins—an update. Molecular bioSystems. 2009;5(4):345–50. 10.1039/b900658n . 19396370

[42] Magalhaes V, Andrade EB, Alves J, Ribeiro A, Kim KS, Lima M, et al Group B Streptococcus hijacks the host plasminogen system to promote brain endothelial cell invasion. PLoS One. 2013;8(5):e63244 10.1371/journal.pone.0063244 23658816 PMC3642152

[43] Seifert KN, McArthur WP, Bleiweis AS, Brady LJ. Characterization of group B streptococcal glyceraldehyde-3-phosphate dehydrogenase: surface localization, enzymatic activity, and protein-protein interactions. Canadian journal of microbiology. 2003;49(5):350–6. 10.1139/w03-042 . 12897829

[44] Henningham A, Chiarot E, Gillen CM, Cole JN, Rohde M, Fulde M, et al Conserved anchorless surface proteins as group A streptococcal vaccine candidates. Journal of molecular medicine. 2012;90(10):1197–207. 10.1007/s00109-012-0897-9 . 22527883

[45] Ling E, Feldman G, Portnoi M, Dagan R, Overweg K, Mulholland F, et al Glycolytic enzymes associated with the cell surface of Streptococcus pneumoniae are antigenic in humans and elicit protective immune responses in the mouse. Clinical and experimental immunology. 2004;138(2):290–8. 10.1111/j.1365-2249.2004.02628.x 15498039 PMC1809218

[46] Dinis M, Tavares D, Veiga-Malta I, Fonseca AJ, Andrade EB, Trigo G, et al Oral therapeutic vaccination with Streptococcus sobrinus recombinant enolase confers protection against dental caries in rats. The Journal of infectious diseases. 2009;199(1):116–23. 10.1086/594372 . 18956975

[47] Feng Y, Pan X, Sun W, Wang C, Zhang H, Li X, et al Streptococcus suis enolase functions as a protective antigen displayed on the bacterial cell surface. The Journal of infectious diseases. 2009;200(10):1583–92. 10.1086/644602 . 19848587

[48] Barrow P. Developmental and reproductive toxicity testing of vaccines. J Pharmacol Toxicol Methods. 2012;65(2):58–63. Epub 2012/01/12. S1056-8719(11)00322-4 [pii] 10.1016/j.vascn.2011.12.001 . 22233769

[49] Palmeira P, Quinello C, Silveira-Lessa AL, Zago CA, Carneiro-Sampaio M. IgG placental transfer in healthy and pathological pregnancies. Clin Dev Immunol. 2012;2012:985646 Epub 2012/01/12. 10.1155/2012/985646 22235228 PMC3251916

[50] Knezevic I. Stability evaluation of vaccines: WHO approach. Biologicals: journal of the International Association of Biological Standardization. 2009;37(6):357–9; discussion 421–3. 10.1016/j.biologicals.2009.08.004 . 19729320

